# Supplementary material for: Tracking neurons across days with high-density probes
Source: Nat Methods. 2024 Sep 27;22(4):778–87. doi: 10.1038/s41592-024-02440-1 (PMC11978519; doi:10.1038/s41592-024-02440-1)
Supplement: Supplementary file 1 — Reporting Summary [file 41592_2024_2440_MOESM1_ESM.pdf]

nature portfolio

Corresponding author(s): Enny H. van Beest

Last updated by author(s): 07/12/2024

Reporting Summary

Nature Portfolio wishes to improve the reproducibility of the work that we publish. This form provides structure for consistency and transparency in reporting. For further information on Nature Portfolio policies, see our Editorial Policies and the Editorial Policy Checklist.

Statistics

For all statistical analyses, confirm that the following items are present in the figure legend, table legend, main text, or Methods section.

n/a	Confirmed	
☐	☒	The exact sample size (n) for each experimental group/condition, given as a discrete number and unit of measurement
☐	☒	A statement on whether measurements were taken from distinct samples or whether the same sample was measured repeatedly
☐	☒	The statistical test(s) used AND whether they are one- or two-sided<br>*Only common tests should be described solely by name; describe more complex techniques in the Methods section.*
☒	☐	A description of all covariates tested
☐	☒	A description of any assumptions or corrections, such as tests of normality and adjustment for multiple comparisons
☐	☒	A full description of the statistical parameters including central tendency (e.g. means) or other basic estimates (e.g. regression coefficient) AND variation (e.g. standard deviation) or associated estimates of uncertainty (e.g. confidence intervals)
☐	☒	For null hypothesis testing, the test statistic (e.g. F, t, r) with confidence intervals, effect sizes, degrees of freedom and P value noted<br>*Give P values as exact values whenever suitable.*
☐	☒	For Bayesian analysis, information on the choice of priors and Markov chain Monte Carlo settings
☒	☐	For hierarchical and complex designs, identification of the appropriate level for tests and full reporting of outcomes
☐	☒	Estimates of effect sizes (e.g. Cohen's d, Pearson's r), indicating how they were calculated

Our web collection on statistics for biologists contains articles on many of the points above.

Software and code

Policy information about availability of computer code

Data collection	We have used SpikeGLX (https://billkarsh.github.io/SpikeGLX/) versions released on 20190413; 20190911; 20190912; 20200520; 20201012; 20201024; 20201103; 20230411 for Neuropixels data acquisition.
Data analysis	Analysis were done in Matlab R2023b and R2024. Neuropixels data were sorted using PyKilosort (https://github.com/MouseLand/pykilosort) and Kilosort 4 (https://www.nature.com/articles/s41592-024-02232-7) as indicated in the manuscript. Bombcell was used to define quality metrics of extracted units (https://doi.org/10.5281/zenodo.8172822) and define well isolated units. The rest of the analysis is described by this manuscript and can be found on Zenodo (https://zenodo.org/records/12734237) and Github (https://github.com/EnnyvanBeest/UnitMatch/tree/v1.0.0_UnitMatch).

For manuscripts utilizing custom algorithms or software that are central to the research but not yet described in published literature, software must be made available to editors and reviewers. We strongly encourage code deposition in a community repository (e.g. GitHub). See the Nature Portfolio guidelines for submitting code & software for further information.

Data

Policy information about availability of data

All manuscripts must include a data availability statement. This statement should provide the following information, where applicable:

- Accession codes, unique identifiers, or web links for publicly available datasets
- A description of any restrictions on data availability
- For clinical datasets or third party data, please ensure that the statement adheres to our policy

Data for mouse ID 1-5 (Extended Data Table 1) are available via figshare (DOI: 10.6084/m9.figshare.24305758.v1) as part of the software demo. Further data is available via figshare (https://doi.org/10.5522/04/24411841.v1), and the rest can be made available upon request.

Human research participants

Policy information about studies involving human research participants and Sex and Gender in Research.

Reporting on sex and gender	N/A
Population characteristics	N/A
Recruitment	N/A
Ethics oversight	N/A

Note that full information on the approval of the study protocol must also be provided in the manuscript.

Field-specific reporting

Please select the one below that is the best fit for your research. If you are not sure, read the appropriate sections before making your selection.

☒ Life sciences ☐ Behavioural & social sciences ☐ Ecological, evolutionary & environmental sciences

For a reference copy of the document with all sections, see nature.com/documents/nr-reporting-summary-flat.pdf

Life sciences study design

All studies must disclose on these points even when the disclosure is negative.

Sample size	We have included data from 25 mice in this study. Specifics can be found in Table S1 of the manuscript. We used the data of five mice to build the method which is described in this manuscript. These data we have already published (https://doi.org/10.6084/m9.figshare.24305758.v1). Subsequently, we validated the method with the remaining data that we had recorded in the lab, and we have received feedback from other users that the described method works for their data as well.
Data exclusions	We included all available chronic datasets we had in the lab at the moment of performing the analysis. Exclusion criteria were based on individual recording quality: a minimum recording time (10 minutes); a minimum number of well isolated units (25 units); a minimum number of matches between recordings (20 matches).
Replication	Everyone can download the software and run the demo (https://github.com/EnnyvanBeest/UnitMatch/tree/v1.0.0_UnitMatch) with example data provided (https://doi.org/10.6084/m9.figshare.24305758.v1) or on their own data.
Randomization	n.a. mice were chronically implanted or not based on the needs for the specific study
Blinding	n.a. we were aware that mice were either chronically implanted or not.

Reporting for specific materials, systems and methods

We require information from authors about some types of materials, experimental systems and methods used in many studies. Here, indicate whether each material, system or method listed is relevant to your study. If you are not sure if a list item applies to your research, read the appropriate section before selecting a response.

Materials & experimental systems

n/a	Involved in the study
☒	☐ Antibodies
☒	☐ Eukaryotic cell lines
☒	☐ Palaeontology and archaeology
☐	☒ Animals and other organisms
☒	☐ Clinical data
☒	☐ Dual use research of concern

Methods

n/a	Involved in the study
☒	☐ ChIP-seq
☒	☐ Flow cytometry
☒	☐ MRI-based neuroimaging

Animals and other research organisms

Policy information about studies involving animals; ARRIVE guidelines recommended for reporting animal research, and Sex and Gender in Research

Laboratory animals	Mice of Bl6 background. 3-9 months of age at implantation surgery. Implanted for max. 6 months.
Wild animals	No wild animals were used in the study.
Reporting on sex	Both males and females were used in this study. For this study sex is irrelevant.
Field-collected samples	No field collected samples were used in the study.
Ethics oversight	Experimental procedures were conducted at UCL according to the UK Animals Scientific Procedures Act (1986) and under personal and project licenses released by the Home Office following appropriate ethics review.

Note that full information on the approval of the study protocol must also be provided in the manuscript.
